# From shallow to deep: some lessons learned from application of machine learning for recognition of functional genomic elements in human genome

**DOI:** 10.1186/s40246-022-00376-1

**Published:** 2022-02-18

**Authors:** Boris Jankovic, Takashi Gojobori

**Affiliations:** 1 Computational Bioscience Research Center, King Abdullah University of Science and Technology, Thuwal, Saudi Arabia; 2 Division of Biological and Environmental Sciences and Engineering, King Abdullah University of Science and Technology, Thuwal, Saudi Arabia

**Keywords:** Genomics, Genomic signals, Artificial intelligence, Sequence analysis, Machine learning, Deep learning

## Abstract

Identification of genomic signals as indicators for functional genomic elements is one of the areas that received early and widespread application of machine learning methods. With time, the methods applied grew in variety and generally exhibited a tendency to improve their ability to identify some major genomic and transcriptomics signals. The evolution of machine learning in genomics followed a similar path to applications of machine learning in other fields. These were impacted in a major way by three dominant developments, namely an enormous increase in availability and quality of data, a significant increase in computational power available to machine learning applications, and finally, new machine learning paradigms, of which deep learning is the most well-known example. It is not easy in general to distinguish factors leading to improvements in results of applications of machine learning. This is even more so in the field of genomics, where the advent of next-generation sequencing and the increased ability to perform functional analysis of raw data have had a major effect on the applicability of machine learning in OMICS fields. In this paper, we survey the results from a subset of published work in application of machine learning in the recognition of genomic signals and regions in human genome and summarize some lessons learnt from this endeavor. There is no doubt that a significant progress has been made both in terms of accuracy and reliability of models. Questions remain however whether the progress has been sufficient and what these developments bring to the field of genomics in general and human genomics in particular. Improving usability, interpretability and accuracy of models remains an important open challenge for current and future research in application of machine learning and more generally of artificial intelligence methods in genomics.

**Supplementary Information:**

The online version contains supplementary material available at 10.1186/s40246-022-00376-1.

## Background

Correct identification of functional elements in the genome forms a crucial step in the process of the functional annotation of genomes. For example, identifying translation initiation sites (TIS) as a starting point of a gene translation is obviously important for the functional annotation of genomes. The presence of such elements is typically signaled by a specific signal motif (also referred to as a marker) or a region in a genome (GSR). It would be beneficial if some kind of rules could be established to help locating GSRs in a genome. There were several reasons for this endeavor, but historically the most important were the cost and efforts associated with experimental identification of GSR. Therefore, if a method could be found that would at the very least reduce the amount of experimental work, this would obviously represent a major step toward efficient functional annotation. Perhaps even more importantly, if additional insights into biochemical mechanisms could be elucidated from such work, that would improve our understanding of molecular mechanisms governing transcription and other cellular processes. Certain statistical rules were observed early on for some genomic signals. For example, one such rule is the Kozak rule [1] in the context of identification of TIS. Although often useful indicators of the possible presence of GSRs, such rules are typically neither necessary nor sufficient and, as such, not a reliable predictor of the presence of GSR. Despite these limitations, observed rules can often lead to reasonable estimates of locations of GSR, particularly when multiple observed rules are combined. The more such rules can be incorporated into prediction methods, the more likely identification of GSRs would be successful. For example, the inclusion of secondary structures into models as well as epigenetic information have all contributed to more powerful prediction models.

However, despite all this added complexity, GSR predictions often turn out to be inadequate. This is principally a natural consequence of the complexity governing cellular processes. The goal of creating a comprehensive model of a cell that explains all experimental observations continues to be elusive.

Therefore, rather than trying to approach GSR prediction problem from “first principles,” the search for GSR prediction models continued in a different direction. Since artificial intelligence (AI) proved to be able to capture complex relationships quite well in a variety of applications, it was no wonder that AI methods become a prominent part of an analytical toolset at the disposal of computational biology. The domain of their applications in biology is very wide and varies from representing reasoning over domain knowledge [2], identification of GSR and many others. Within the framework of AI methodology, the Machine Learning (ML), described in more detail in the next section, became the dominant approach for signals recognition in the genome.

These methods leveraged the already known rules related to specific genomic signal locations and combined them with additional rules deemed potentially useful. Also, the growing number of experimentally verified signal locations in a wide variety of organisms allowed for a more systematic study of factors that may be involved in gene regulation, transcripts, translation, and other cellular processes.

First and foremost, the question is how well do ML models capture the cellular mechanisms involved? This is usually judged by the predictive ability of a model: given a set of unknown genomic sequences, can such models accurately identify a GSR of interest?

In the simplest (and most widely studied case), the motif associated with GSR is known a priori. For example, translation is predicated by the presence of an AUG codon in RNA, while the presence of AG/GU would indicate potential intron/exon boundaries. In such cases, the task of predicting a location of a GSR reduces to the one of binary classification. In other words, given a genomic sequence and given a marker (signal) for the GSR within that sequence, the task is to simply pronounce whether a marker at a specific location in the genome represents a true or false GSR signal. In its most basic form, functional annotation means locating in the genome positions of important functional elements, such as TIS and alternative splicing positions. If, for example, an AUG codon is known to be a TIS in RNA, the corresponding ATG codon in the primary genomic sequence is marked as a TIS site in the functionally annotated genome.

Such a prediction approach can obviously work in situations when we know what we are looking for. However, when signals are not well defined, such as in the case of cis-regulatory regions, this a strategy cannot be used, and different modeling approaches must be employed, resulting in more complex prediction models.

The success of ML models depends on both the quality and quantity of data used to develop and test the models, as well as the mathematical and computational sophistication of the model. Over time, great advances were made on both counts. As far as data are concerned, the next-generation sequencing technologies (NGS) resulted in an exponential increase in the volume of genomic and other OMICS data. Not only that, the quality, meaning the correctness, has also steadily improved. This is of course beneficial, but it is the corresponding increase in computational capacity and high-performance computing that allowed for the processing of very large volumes of data. Finally, the models themselves evolved to be more sophisticated, resulting in them being better at capturing the underlying molecular mechanisms.

The question is, how much improvement do novel models bring to this analysis? In this paper, we surveyed many of these methods and looked into what can be learned from this evolution in modeling. In doing so, we present, among others, some of our experiences in applying various ML models to similar or identical problems, compare the model performances and try to identify where possible improvements may have come from. Some aspects of model evolution from classical, “shallow” ML models toward deep learning (DL) models are also discussed. Finally, we look into tradeoffs between simpler and more complex models when used in the field of genomics.

In this review, we address the evolution of ML modeling when applied to the identification of a set of GSR in the human genome, namely TIS, splice sites, polyadenylation signals and enhancer regions. Although we are primarily interested in the performance metrics of the models, there are some other very important aspects of these models, such as their interpretability. This important and complex subject is left for a future review.

## ML and genomics

The basic hypothesis most commonly used when developing GSR locations prediction models is that information used by the transcription machinery is contained somewhere in the surrounding sequences of the signal. This is not a completely accurate presentation of reality. Numerous other factors are known to have an impact, and these are subject to distant cis-regulatory regions, histone modifications, etc. Nevertheless, since we focus here mainly on the evolution of predictive models, epigenetic effects will not be specifically addressed, even though we acknowledge that not taking them into account has an impact on the resulting models and their utility.

ML models in genomics are almost always derived through supervised learning. In supervised learning, ML models are given inputs and the corresponding outputs explicitly. Based on that information, the model captures the relationship between inputs and outputs, or more generally, causes and consequences (see, for example [3]). In the case of identification of GSR, a subset of experimentally verified true and false signals and their corresponding surrounding sequences are used to train ML model. These models are then applied to an unused portion of the dataset (i.e., those not used for model training), and some measure of model accuracy is derived. These measures are then used to compare the predictive power of one model to another; however, direct comparisons are often tricky as they are context and data-dependents. But there can be no doubt that over time the predictive power of models has been improving, as the analysis of surveyed work further in this text implies.

What we refer to as “shallow ML” models are the models, such as artificial neural networks (ANNs) or support vector machines (SVMs) that rely on incorporating prior information about the phenomena that is modeled. They require expert domain knowledge and are therefore approachable only by a few. In a sense, this approach resonates with the “first principles” methods in that some relationship between causes and consequences can be inferred or at least suspected. The prior knowledge about a process modeled is incorporated through a set of features, which represent inputs to such models. The features used depend on the problem at hand and will be revisited in more detail and specific examples later in the text. The principal problem with this approach is that there is little reliable guidance in the selection of relevant features as many of the molecular and biochemical processes involved in genomic signaling are not well understood. Because of this uncertainty, one approach is to overprovision the number of features in the hope that the less important ones would be eliminated during the model training. This is however far from ideal as it can result in more complex and often overtrained models.

Deep learning (DL) models, on the other hand, rely on the ability of models to extract characteristics (features) of data by the model itself, thus reducing the reliance on prior knowledge and supplied features. This is especially important when modeling phenomena that are not well understood and with little guidance as to what would constitute a reasonable feature set. However, as there is no such thing as a free lunch, DL models require complex structures as well as very large datasets in order to train them. DL methods have resulted in some truly outstanding successes such as image recognition, natural language processing, games, etc. (described in, for example, [4–6]). In all such cases, however, the models were trained on enormous datasets, sometimes with millions of labeled data points and some very complex and deep structures, requiring long training on high-performance computers. These requirements are seldom met in the case of genomic signal analysis, so the question is what DL models or, more precisely DL-like models can bring to genomic sequence analysis. In many cases, the models employ some DL techniques, but not to the same extent as in the case of larger DL models.

### Models and features

Perhaps the single most important decision the builders of prediction tools must face is the selection of model features, especially when dealing with shallow machine learning models. Entire information content that is supplied to the model is done via features of data on which the model is trained and used. Since the model cannot take a sequence of nucleic acids directly as an input, such sequence is represented to the model as a vector of so-called model features, each representing a specific aspect of the input sequence. Features can take many forms, and there are no fixed rules on how to select them. Domain knowledge and experience of the modeler are perhaps the best guiding principles.

In the case of genomic signaling, these features could be sequential (presence of certain motifs) or statistical (position weight matrices of specific regions, the relative frequency of nucleotides, dinucleotides, trinucleotides), etc. In addition, various physicochemical, structural, and thermodynamic properties of nucleotides or nucleotides groups can also be used as features [7–11]. An interesting example of a feature set can be found in [12], Supplementary Material 2. Of 274 features in the feature set, 110 represent features based on physicochemical properties, while the remaining 164 are statistical features were derived from nucleotide sequences. Such a large number of features is partially the result of insufficient knowledge as to the effects features may have. A large number of features is generally undesirable: it leads to complex models that are prone to overfitting. Therefore, simple, parsimonious models are more desirable as they tend to be more robust when applied to unseen datasets. This problem, when related to GSR prediction, is discussed, for example in [13, 14]. Feature selection is an important step in modeling to address the tradeoff between the desired model simplicity and the uncertainty as to which of the many possible features are relevant. Feature selection aims at discarding as many redundant, unnecessary features as possible without impacting the accuracy of the model. One commonly used method is to reduce the dimensionality of the feature vector by looking into various measures of correlation between features and eliminating those that bring little new information. When computational resources allow, a number of subsets from the initial feature set can be tried, although this presents a combinatorial problem.

Theoretically, many of these problems would not present themselves when DL approach is used, as features are extracted automatically during training. However, this requires a very large amount of training data and is not suitable for many problems in GSR recognition. Nevertheless, some more recent models were built based on the DL approach and are described further in the text. Some combination of DL and ML modeling approaches by incorporating known features (prior information) can also be used.

In summary, the complexity of models is largely driven by the dimensionality of data and the depth of ANNs and some compromises must be made. In order to investigate how well the application of DL models works on genomic signaling problems, we present an overview of some common problems tackled by both ML and DL modeling and see what, if any, conclusions can be drawn from them.

## Methods

**Fig. 1:**
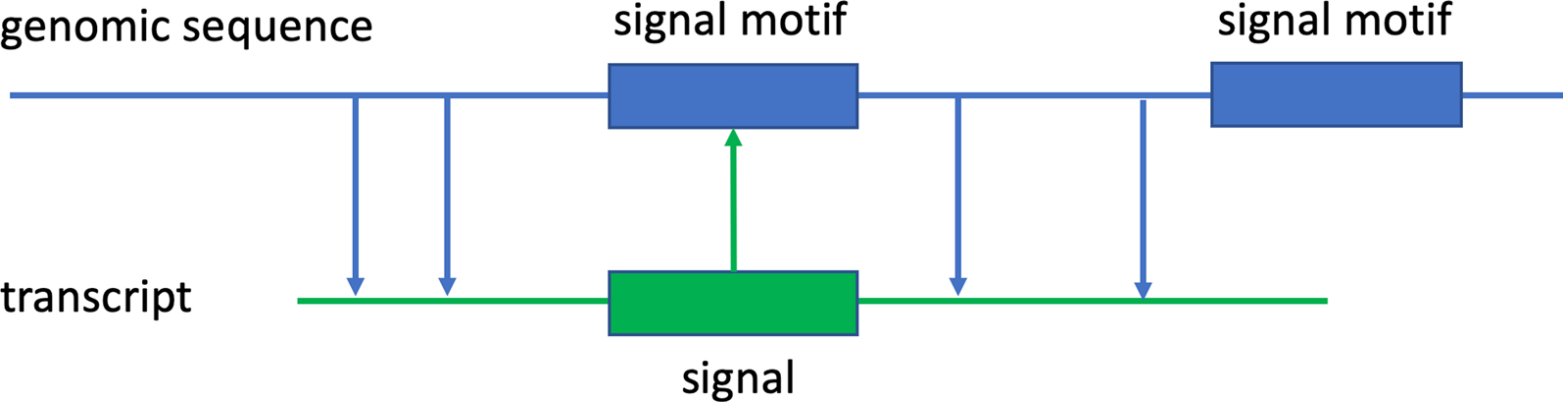
Relationship between true signals and signal motifs

In all cases studied, we are concerned with the problem of recognizing GSR in primary genomic sequence. That means the location in the genome of the GSR that is transcribed or later translated into the actual signal used in the cell nucleus. This concept is graphically represented in Fig. 1. Although more than one signal motifs are present in the figure, and they are both candidate signals, only one of them is translated and should be recognized as a true site. The other one should be identified as false by the prediction tools. For example, an ATG signal motif may be translated into a true TIS in the transcripts, in which case we would consider such ATG signal motif as a “true” signal; otherwise, it is considered “false.”

Clearly, successful recognition of such motifs (as opposed to signals in the transcripts) requires high specificity as signal motifs are typically abundant in a genome. In the case of TIS, there are approximately 50 million ATG motifs in the human genome, but less than one percent of those are translated into TIS.

In most cases, new results are published with a claim that the model provides better results than those previously reported. However, independently verifying these claims is difficult. This is particularly the case for earlier works where often no prediction tools or programs were provided, thus making it impossible to independently verify model performance claims.

In order to provide more unbiased comparisons, in addition to the results reported by the authors, we use the performance results obtained when such models were retested on datasets other than those originally used for model training whenever reported in the literature. This is often the case in more recent works where authors often provide performance figures for previously reported tools (when available) that are retested on the datasets they used. While not perfect, these tests nevertheless provide an important additional insight into the behavior of models when applied to previously unseen datasets. In cases where no tools are provided to independently verify the performance claims (this is sometimes the case in earlier works), we use the figures that the authors provided, although our experience is that such figures should be treated with caution. In general, claimed performances of those early models are almost always too optimistic.

Importantly, different performance metrics are reported in the literature, making further complications in comparisons. The question of which performance metric represents the best description of model performance is a complex one and is often driven by the intended use of the performance figures. In this study, our preferred performance measures are sensitivity, specificity, and accuracy, as they are good descriptors of the actual prediction tool. Sensitivity (Se) represents the proportion of correctly predicted true signals (true positives, TP), specificity (Sp) represents the proportion of correctly predicted false signals (true negatives, TN) and accuracy (Acc) represents the proportion of correctly predicted samples out of all samples. These metrics are calculated as follows:

$${\text{Se}} = \frac{{{\text{TP}}}}{{{\text{TP}} + {\text{FN}}}};\,{\text{Sp}} = \frac{{{\text{TN}}}}{{{\text{TN}} + {\text{FP}}}};\,{\text{Acc}} = \frac{{{\text{TP}} + {\text{TN}}}}{{{\text{TP}} + {\text{TN}} + {\text{FP}} + {\text{FN}}}}$$

where FN and FP represent false negatives and false positives, respectively.

However, due to the variety of reported performance evaluation metrics, not all surveyed work is included in the comparison study. We take into account only those prediction tools for which, at minimum, either the sensitivity and specificity pair or the accuracy are reported. As a consequence, certain entries in performance metrics given in Tables 1, 2 and 3 later in the text are with no value assigned.

**Table 1:** Performance of TIS prediction models (*Se* sensitivity, *Sp* specificity, *Acc* accuracy)

Tool	Reference	Year	Results		
			Se	Sp	Acc
Pedersen and Nielsen	[15]	1997	65	82	
Salzberg	[16]	1997	74	68	
Zien et al	[14]	2000	76	78	
Zeng et al	[13]	2002	76	94	85
Pertea and Salzberg	[17]	2002			84
Sayes et al	[18]	2007	80	81	
Tikole	[19]	2008	83	73	74
iTIS-PseTNC	[20]	2014			78
TITER	[21]	2017	81	90	85
DeepGSR	[22]	2018			94
Goel et al	[3]	2020	77	98	97

Entries with no value are explained in “Methods” section

**Table 2:** Performance comparison for acceptor and donor site locations prediction; *Se* sensitivity, *Sp* specificity, *Acc* accuracy

Tool	Reference	Year	Signal type	Se	Sp	Acc
GeneSplicer	[24]	2001	Acceptor	69	97	83
			Donor	60	98	79
SplicePredictor	[25]	2004	Acceptor	84	92	88
			Donor	79	97	88
Zhang	[26]	2010	Acceptor	90	90	
			Donor	93	93	
Bari	[27]	2012	Acceptor	77	89	89
			Donor	89	97	95
Goel	[23]	2015	Acceptor	94	94	
			Donor	91	94	
Wen	[29]	2017	Acceptor			93
			Donor			92
DeepSS	[30]	2018	Acceptor			95
			Donor			95
SpliceRover	[31]	2018	Acceptor	91	97	95
			Donor	90	96	96
Splice2Deep	[32]	2020	Acceptor	98	95	97
			Donor	99	96	97

Entries with no value are explained in “Methods” section

**Table 3:** Performance evolution of poly(A) tail prediction models (*Se* sensitivity, *Sp* specificity, *Acc* accuracy)

Tool	Reference	Year	Adjusted values		
			Se	Sp	Acc
Polyadq	[36]	1999	46	86	65
PolyA Signal Miner	[37]	2003	72	80	
ERPIN	[38]	2003	66	88	75
PolyA_SVM	[39]	2006	56	78	68
PolyFd/PolyFud	[40]	2009	72	80	78
Polyapred	[41]	2009	57	86	
Polyar	[42]	2010	57	50	53
Chang et al	[43]	2011	56	90	75
DPS-ANN	[12]	2012			78
HMM-SVM	[44]	2013	80	87	81
DSET	[45]	2015	86	86	86
Omni_PolyA	[35]	2018			80
DeepGSR	[22]	2019			84
DeeReCT-PolyA	[46]	2019			84

Entries with no value are explained in “Methods” section

For some predictive tools, it was possible to reconstruct our preferred performance metrics from raw results data even though the authors did not specifically calculate them. Finally, in some cases where neither raw data nor preferred performance measures were reported, we relied on the results when such tools were independently tested in other peer-reviewed works on different datasets. Whenever a tool was tested on several datasets, adjusted performance metrics were used for the purpose of comparison. Adjusting would typically make use of averages or weighted averages of compatible performance metrics, depending on the test dataset sizes (when this information is available). Detailed reported, retested and adjusted performance figures for all predictive tools analyzed are given in the Supplementaries for each GSR type.

It should be noted that models were trained and tested on different datasets, which, over time, become larger and of better quality. The AI and ML theory, as well as computational capacity, have also improved over time. Taking all this together, it is clear that precise comparison of models from different epoch is imprecise; nevertheless, studying the evolution of models is useful in order to see what lessons can be learnt and what can be realistically expected in the future.

## Case studies

In this review, we focus on four commonly studied functional elements/regions and the corresponding recognition models. The signals in question are TIS, alternative splicing sites, polyadenylation sites, and enhancers in the human genome. These GSR are selected because the application of ML in their recognition has been extensively studied over lengthy period of time. Also, the number of models reported in literature offers a unique opportunity to evaluate the progress made in the application of ML to recognition of functional elements in the human genome.

What the first of the three problems mentioned above have in common is that the candidate signals are well defined (though in the case of polyadenylation, this is only slightly more complicated). In such cases, a predictive model is a binary classifier that whenever presented with a well-defined candidate signal motif pronounces a verdict. Predicting cis-regulatory elements is an evolving science; they are not as well defined, and approaches used in the case of the previous three cases cannot be directly replicated in this case.

### Case 1: translation initiation sites (TIS)

The problem at hand is to recognize locations in the human genome that are translated into the start codon in RNA, following the pattern introduced shown in Fig. 1. As stated, it is a well-defined problem, and for that reason it is one of the early problems addressed with ML. Since it is known that such sites are located at ATG codon position in a genome, the problem reduces to a classification problem—is a given ATG trinucleotide in the genome translated into a TIS in RNA or not. A typical classification methodology would take as an input a set of true ATGs (in the sense that they are translated into a start codon) together with the surrounding sequences in both 5’ and 3’ directions. These surroundings are often but not always selected symmetrically around the ATG trinucleotide. For example, it could be argued that more weight should be given to 5’ side data as the promoter region is located there. Different lengths of the surrounding sequences are used, varying from tens to the hundreds of base pairs in either direction. These sequences with true signal motifs comprise the positive dataset. The negative dataset is formed in the same manner and in the same sequence format. The only distinction is that in the negative dataset, the ATG trinucleotides are confirmed negative, i.e., they do not correspond to a start codon.

Data for model training and testing are nowadays easy to obtain with an increase in the number of functionally annotated genomes with indicated locations of TIS sites in the chromosomes.

Regardless of the type of ML model used, in order to classify a candidate signal, information contained in the surrounding sequence must be provided to the model as input in some form. The actual prediction model does not work with sequences directly but with a number of representations of these sequences. For example, one input into the model could be the frequency of adenine in the 5’ region from the ATG motif. For each tool, these features are described in detail in the corresponding reference.

**Fig. 2:**
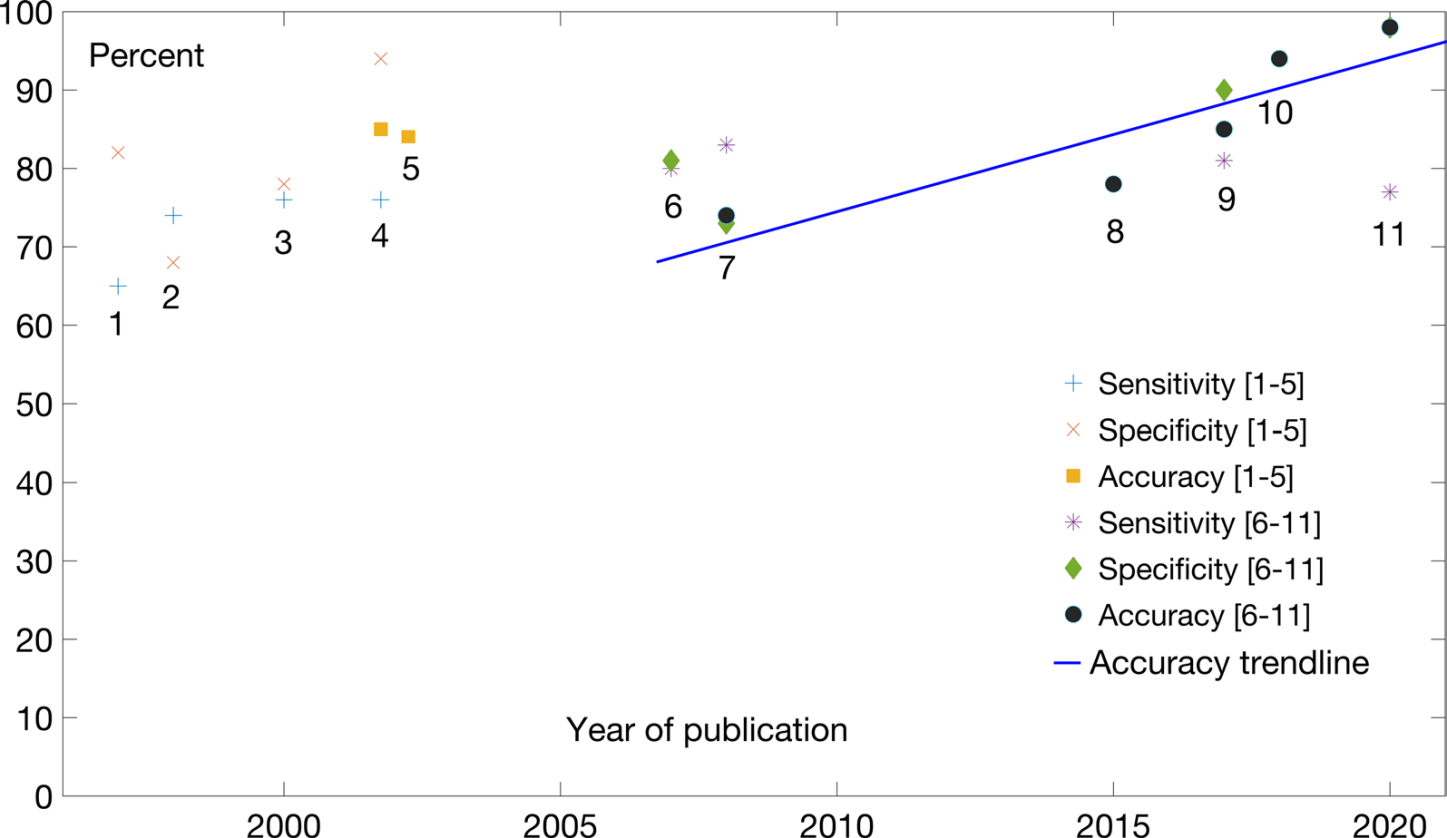
Performance of TIS prediction models

We surveyed a number of TIS recognition models applied to the human genome and present reported and adjusted performance metrics. Details of the performance figures are given in Supplementary 1. The adjusted performance metrics are plotted in Fig. 2 and also given in the tabular form in Table 1.

The models from Fig. 2 are as follows: (1) Pedersen and Nielsen used ANN to identify correct AUG codon in mRNA [15]; (2) Salzberg’s predictor for both TIS and splice sites [16]; (3) Zien et al*.* used SVM for prediction of TIS [14]; (4) Zeng et al*.* model used seven features for TIS prediction in [13]; (5) the model from Pertea and Salzberg [17]; (6) Sayes et al*.* experimented with different models complexity in [18]; (7) Tikole and ﻿Sankararamakrishnan used ANNs for prediction in [19]; (8) Chen et al*.* used the physicochemical properties and pseudo trinucleotide compositions as features in [20]; (9) Zhang et al. developed TITER, a DL model in [21]; (10) Kalkatawi et al. developed DeepGSR, a DL model in [22]; (11) Goel et al. developed an SVM-based model in [23].

The accuracy trendline in Fig. 2 refers to tools listed in 6–11 above. The tools listed in 1–5 were not generally tested on primary genomic data but on RNA or cDNA data. They are nevertheless included for historical context. The accuracy trendline is purely illustrative; as in the case of other GSRs analyzed later in the text, the progress in accuracy improvement is not linear. However, drawing of a trendline is useful in the sense that the evolution in improvements becomes noticeable. Of course, models reported are usually claimed to have made an improvement in performance over the previously reported models, although such claims do not always stand up to scrutiny after being retested independently. Nevertheless, there is a noticeable improvement in performance that is due to both improvements in data and ML methods.

Models reported in [21, 22] are developed using some methods from the DL toolkit: they are not as reliant on features selection as the “shallow” models and use certain other approaches from DL, such as max-pooling and convolution.

It should also be mentioned that more TIS recognition tools have been developed for non-human genomes, although they are not analyzed in this study.

### Case 2: identification of splice sites

Another problem often addressed with ML methods is the recognition of alternative splice sites. The objective is to locate the sites in human genome that correspond to alternative splice sites in the resulting transcripts. This is a different problem to the one of identifying splice sites solely in RNA sequences, although so derived models can also be used in locating the sites in primary genomic sequence.

Identification of splice sites is similar to the TIS recognition problem in that the candidate signals GT/AG (that correspond to GU/AG donor and acceptor sites at intron boundaries in RNA) are well-defined.

**Fig. 3:**
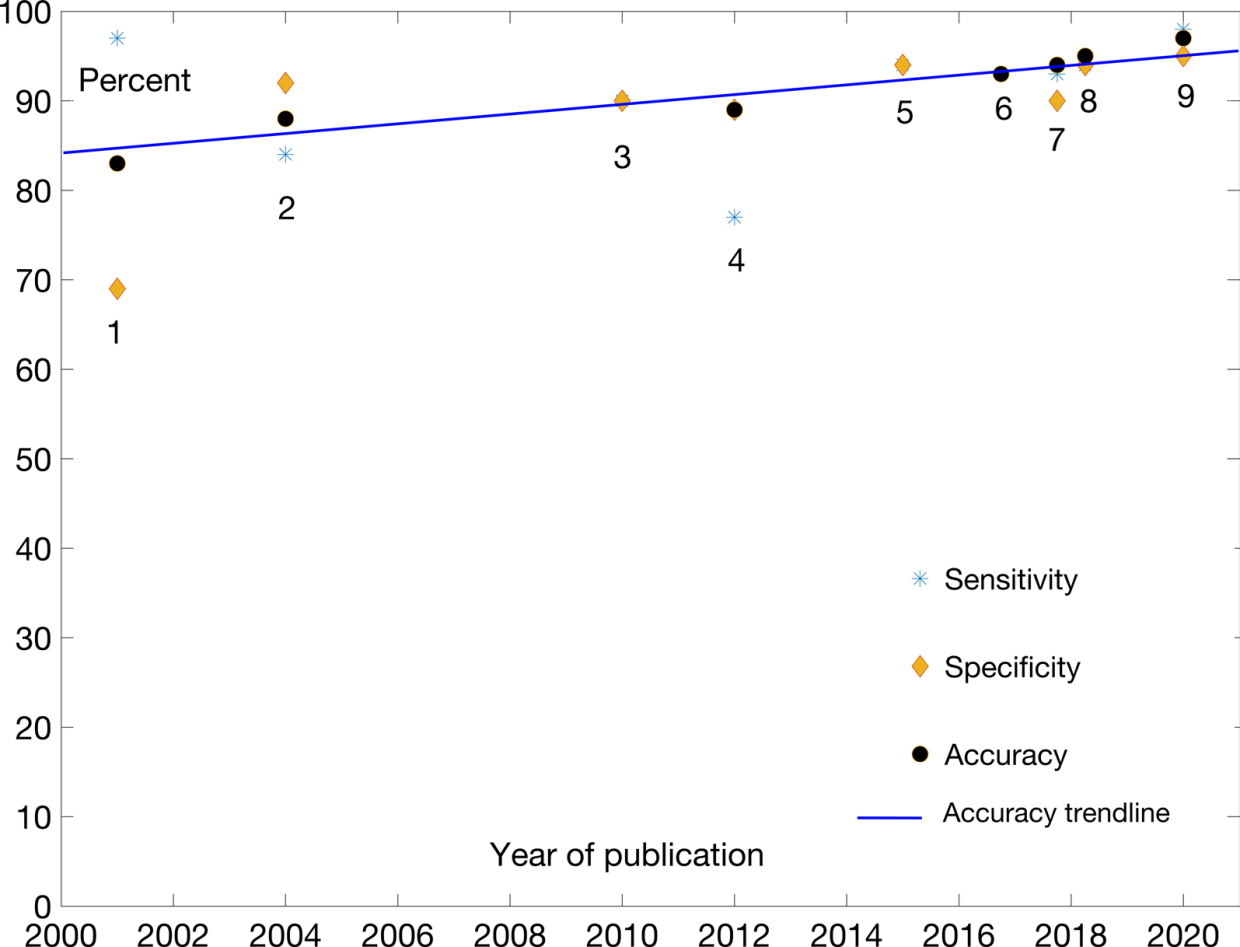
Performance comparison for acceptor site locations prediction

**Fig. 4:**
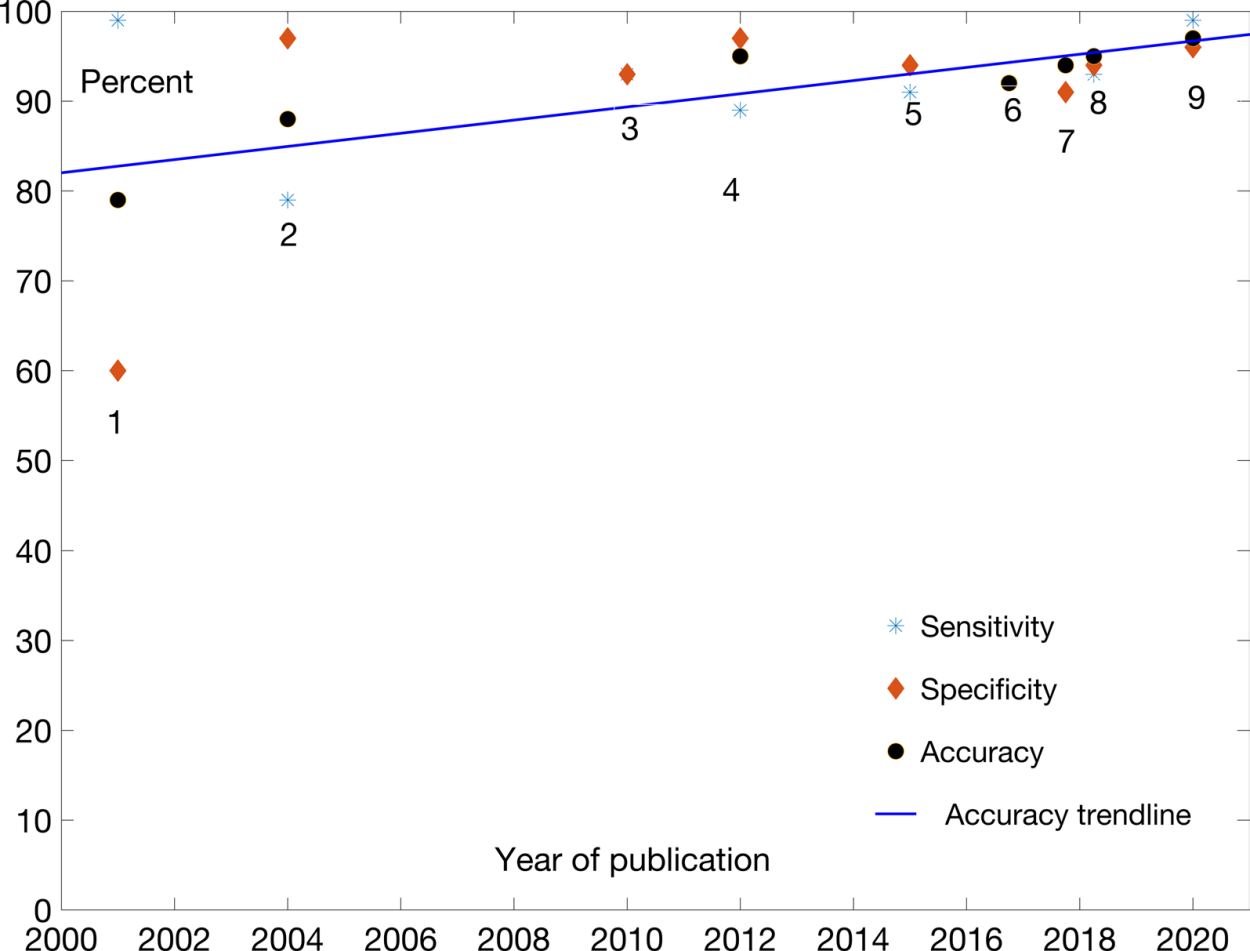
Performance comparison for donor site locations prediction

The performance results are typically reported separately for acceptor and donor sites. The tools surveyed here are as follows: (1) GeneSplicer [24]; (2) SplicePredictor [25]; (3) Zhang et al*.* [26]; (4) *Bari *et al*.* [27]; (5) Goel et al*.* [28]; (6) Li et al*.* [29]; (7) DeepSS [30]; (8) SpliceRover [31]; and (9) Splice2Deep [32]. Figures 3 and 4 graphically represent the evolution of models’ performance for acceptor and donor sites, respectively. The results are also given in the tabular form in Table 2. Details of the reported and retested prediction tools performance data are given in Supplementary 2.

Generally speaking, similar features are used in this case as in the case of TIS. Again, the progress has not been linear, but the accompanying trendlines are nevertheless useful as an illustration of improvements. DeepSS, SpliceRover and Splice2Deep are more recent tools built with some DL features. They provide improvements in recognition accuracy, although not spectacularly so when compared to the previous, “shallow” models. This is likely related to limited information content present in linear sequence representation of genomic sequences used in model training. Both modeling approaches seem to be able to extract such limited content.

### Case 3: identification of poly(A) signals

Polyadenylation is the process in eukaryotic organisms of appending long sequences of adenines, referred to as the poly(A) tail, at the end of the primary transcripts after cleaving. The primary purpose of the poly(A) tail is to stabilize the RNA molecular chain, which is important for the integrity of further processing in the nucleus [33]. The location of the tail is preceded by poly(A) signal (PAS). There are 12 variants of motifs in humans that signal a poly(A) tail. A PAS signal motif is a sequence of six nucleotides. Moreover, the distance between a PAS and poly(A) tail start location is not fixed, but subject to certain distributions, that in themselves are unique to each PAS motif type. The task here is to determine a location in the primary genomic sequence corresponding to the location of the PAS in transcript. This is different from the problem of identifying the actual PAS in RNA, which is also studied in the literature.

Recognition of PAS sites is a more complex problem than those described in the two previous cases. Constructing a training data set is a challenge as PAS motifs come in at least 12 variants in humans. PAS motifs and the associated distributions in distances between PAS and poly(A) tail start in humans are comprehensively studied and summarized well in [34]. Most strategies deployed to construct labeled datasets relied in one way or another on such distributions. The construction methods of these sets are somewhat complicated and not specifically discussed here, but detailed descriptions can be found, for example, in [12, 35]. An additional source of data can be found in GENCODE https://www.gencodegenes.org/human/release_21.html, although it should be noted that the list of poly(A) sites there does not form a part of the main annotation file.

We analyzed the performance of the following 14 human PAS prediction tools: (1) Polyadq [36]; (2) PolyA Signal Miner [37]; (3) ERPIN [38]; (4) PolyA_SVM [39]; (5) PolyFud [40]; (6) Polyapred in [41]; (7) Polyar [42]; (8) from [43]; (9) DPS-ANN [12]; (10) DSET [44]; (11) DSET [45]; (12) Omni_PolyA [22]; (13) DeepGSR [22]; and (14) DeeReCT-PolyA.

**Fig. 5:**
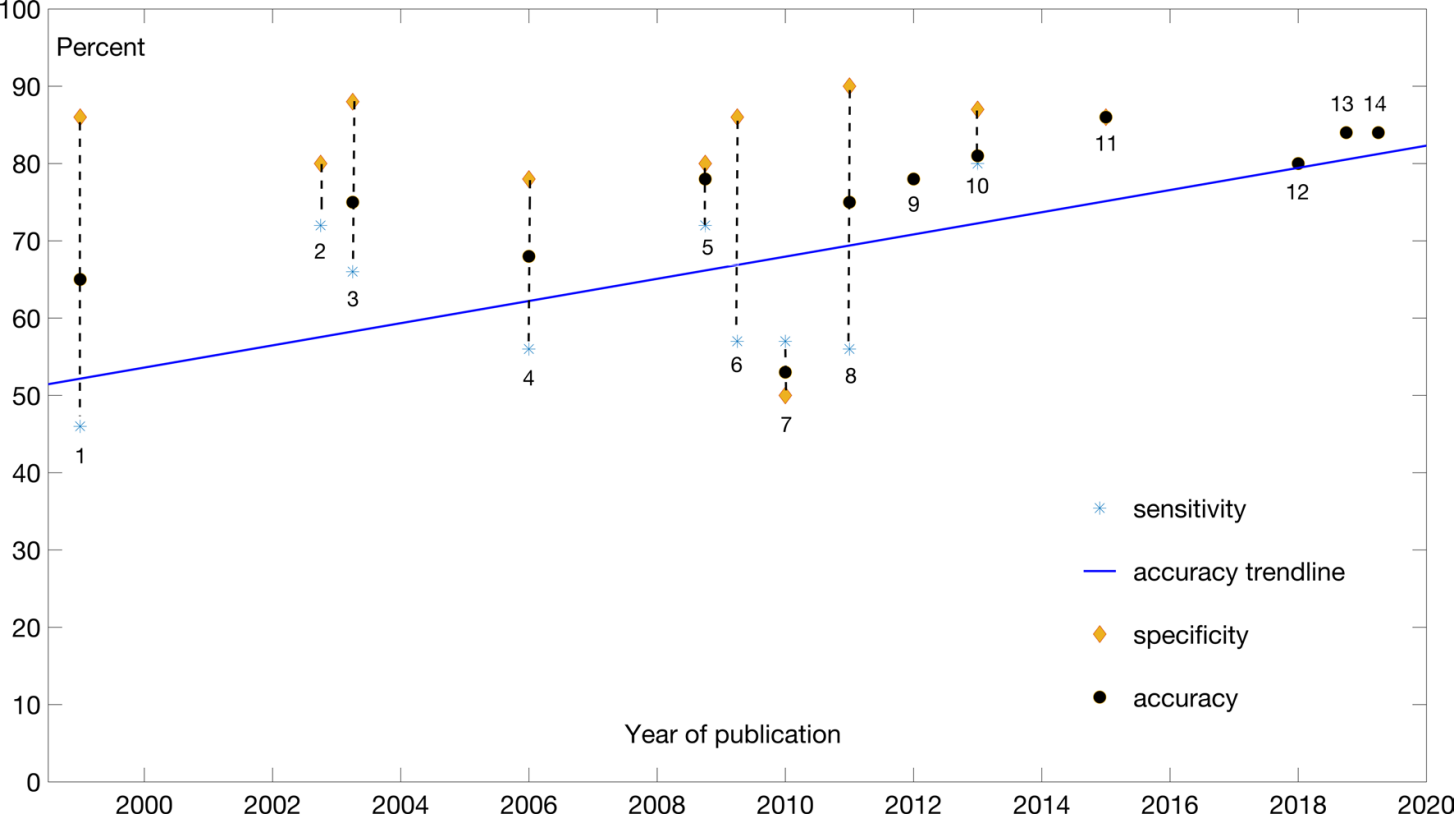
Performance evolution of poly(A) tail prediction models

The evolution of model accuracy is presented in Fig. 5 and in tabular form in Table 3. A detailed description of reported and retested results is given in Supplementary 3, together with the calculated adjusted performances used in the comparison analysis.

It should be noted that some earlier prediction tools are trained only on the most common PAS variant. In cases where tools are trained for all variants, an adjusted aggregate metric is used, as described in Supplementary 3. For that reason, the performance of the tools that are trained only on the dominant PAS variant is strictly speaking not directly comparable to the performance of those models trained on all variants. Nevertheless, since the dominant variant carries the most weight, we believe that including the performance of these tools in comparison is meaningful.

The accuracy of poly(A) tail location prediction has been steadily improving over time. This is both due to an improvement in the quantity and quality of the available datasets as well as improvements in modeling approaches. Several tools based on DL approaches were developed, such as those reported in [22, 35, 46]. These delivered a reasonably balanced performance, although an improvement in models accuracy improvement has been relatively modest. It is possible, though, that with the increase in the amount of data available for modeling, further improvements could be expected from DL models.

### Case 4: enhancers

A different challenge is posed by the identification of regulatory modules in genomes, such as enhancers. Enhancers are regions in the genome that play a role in regulation of gene expression. Unlike the previous cases, there are no simple, fixed signals that an ML classifier can be applied to. Enhancers are typically located at a distance (thousands of base pairs) from the genes whose expression they regulate. They have also been linked to transcription initiation, temporal and tissue-specific gene expression.

In general, the problem of enhancer prediction can be stated as follows: does a given region in a genome (and a tissue type) contain an enhancer. The prediction models have to be able to distinguish an enhancer region from the background sequence. A number of methods have been proposed for the identification of enhancers. We briefly discuss below some ML methods used as well as some model prediction performance matters. An important property of enhancers classes is that they are strongly tissue-specific, and for that reason, the corresponding prediction tools are typically tissue-specific or cell-line specific. Direct comparison of different enhancer prediction models is therefore difficult, but some general conclusions can be made about the utilization of ML and DL models for this problem. It should be noted that prediction is primarily related to the presence of enhancer regions in a given sequence and not to their association with specific target genes. Associations between regulatory elements and genes are complex. An example of such associations between genes and transcription factors is reported in [47]. Some efforts toward modeling such interaction in the case of enhancers are given in [48].

Numerous ML methods have been deployed for this purpose. A comprehensive review of many earlier works in enhancer prediction is given in [49]. A number of enhancer prediction methods have been developed based on epigenetic markers. For example, methods based on the application of Hidden Markov Models (HMMs) to chromatin modification signatures are presented in Won et al*.* [50], in Ernst and Kellis (ChromHMM [51, 52]) and in Won et al. (ChroModule [53]). SVM-derived models are developed in Fernández and Miranda-Saavedra (ChromaGenSVM, [54]), Fletez-Brant (kmer-SVM, [55]) and in Ghandi et al. (gkm-SVM, [56]). Models using ANNs are found in Firpi et al. (CSI-ANN, [57]) and Kleftogiannis et al*.* (DEEP, [58]), although the latter utilizes ANN in addition to SVM.

In all the models listed above, a set of hand-crafted features had to be defined. Both epigenetic and sequence features are used for this purpose. With the emergence of DL methods, new approaches in enhancer prediction became available. Moreover, DL models are typically able to rely on sequence data only, which greatly reduces the complexity of the modeling process.

An example of the DL model applied to enhancer prediction problems is DeepEnhancer, described in [59]. This is a 14-layer convolutional network with max pooling and batch normalization. There are some interesting conclusions in this work, such as that increasing the depth of the ANN (number of layers) can result in a weaker predictive ability of the model. The authors attribute this to an insufficient amount of training data, which illustrates some known limitations in the applicability of DL models.

Two other works in the prediction of enhancers, Chen et al. [60] and by Hong et al. [61], are interesting in that the models derived on several species were tested in a cross-species manner. The important conclusion is that mammalian enhancers are well conserved across species (this is similar to the case of transcription factors reported in [47]). Moreover, this conservation is strongly tissue-related in the sense that the similarity in enhancer sequences is stronger in the same tissues across species than in enhancers within the same organisms but across different tissues. CrepHAN, described in [61] is trained by using hierarchical attention networks, which are typically used in natural language processing (NLP). The entire genome can be considered a set of words of a certain fixed length (i.e., a set of k-mers) and to which word embedding is applied. In the comprehensive study reported in [60], using data from various tissues in humans, mouse, dog, opossum, cow and macaque, an SVM model is used first, where features are constructed from a frequency of 5-mers in the sequence that is classified. In the second part of this work, a convolutional neural network is trained on genomic sequence data. The authors make an interesting observation in that while the SVM model had a somewhat lower ability to distinguish enhancers from background sequences compared to the convolutional network model, it had better cross-species enhancer prediction results. The authors concluded that convolutional models are likely better able to capture certain enhancer features unique to individual datasets, at the cost of losing some ability to aggregate common enhancer properties across species. We would here infer the same conclusion as the authors in [61] regarding the need for more training data when DL models are used. Finally, the authors established a quantitative measure of the similarity of short sequences present in enhancers and in transcription factor binding motifs, which is an interesting result.

## Conclusions

ML applications have produced remarkable results in a number of applications. Image recognition, natural language processing, self-driving cars, games, and many others are examples of progress in the field. It is therefore no surprise that computational biology, and genomics in particular, should be another area that could be revolutionized by ML. The volume of published work in the application of various flavors of ML in genomics reflects this expectation.

We analyzed a set of tools for the identification of four specific GSR that covers a period of more than 20 years. This period has seen dramatic developments and improvements in modeling methods, data analysis, genomic data generation and annotation, and above all, a spectacular increase in computational power and storage capacity. It would be, therefore, reasonable to expect a steady improvement in the performance of models for the prediction of genomic signaling and regions. Reflecting on the results of this survey, it is evident that some progress has been made, but perhaps not entirely in line with expectations. A wide variety of methods, approaches, datasets, features, correction factors, prior knowledge, deep neural networks and other strategies have been tried. The results are mixed. One sign of the progress is that more recent models tend to have a more balanced sensitivity and specificity and more consistent performance when applied to different, previously unseen data. This is in part due to applications of more advanced modeling theory as well as availability of more abundant data that are also of better quality. Computational power has also increased over time, but we do not regard this as crucial due to the relatively modest dataset size involved in this type of modeling. Nevertheless, there seems to be a plateau that model performance has reached and incremental improvements are not large. Does this imply that some limit has been reached in our ability to advance computational prediction of GSR signals? It appears that some visible advances of late works are likely to be attributed to a more elaborate analysis of biochemical and molecular processes and their incorporation into feature sets. Thus, it is reasonable to assume that finding improved and more relevant feature sets would be the main avenue for further refinement of the prediction models. That would also have an added advantage in helping to identify the molecular mechanisms involved.

Although we have not set out to discuss the utility and interpretability of these models here, it can be noted that some results, such as quantification of the level of conservation in regulatory and cis-acting elements are important results achieved through the application of machine learning. In general, however, the interpretability of GSR prediction models remains an open challenge.

## Supplementary Information

**Additional file 1. Supplementary material 1.** **Table 1:** Performance comparison between different genomic TIS location prediction tools. Se denotes sensitivity, Sp specificity and Acc accuracy.

**Additional file 2. Supplementary material 2.** **Table 1:** Performance comparison between splice site prediction tools. Se denotes sensitivity, Sp specificity and Acc accuracy.

**Additional file 3. Supplementary material 3.** **Table 1:** Performance comparison between different poly(A) tail prediction tools. Se denotes sensitivity, Sp specificity and Acc accuracy.

## Data Availability

Not applicable.

## Abbreviations

TIS: Translation initiation site
GSR: Genomic signals and regions
ML: Machine learning
DL: Deep learning
AI: Artificial intelligence
ANN: Artificial neural networks
SVM: Support vector machine
NGS: Next generation sequencing
NLP: Natural language processing
