# Supplementary material for: From shallow to deep: some lessons learned from application of machine learning for recognition of functional genomic elements in human genome
Source: Hum Genomics. 2022 Feb 18;16:7. doi: 10.1186/s40246-022-00376-1 (PMC8855580; doi:10.1186/s40246-022-00376-1)
Supplement: Supplementary file 1 — Additional file 1. Supplementary material 1. Table 1: Performance comparison between different genomic TIS location prediction tools. Se denotes sensitivity, Sp specificity and Acc accuracy. [file 40246_2022_376_MOESM1_ESM.pdf]

SUPPLEMENTARY MATERIAL 1

The table below lists the surveyed genomic TIS location prediction tools and details the reported and reassessed performances.

Tool	Reference	Year	Reported			Adjusted			
			Se	Sp	Acc	Se	Sp	Acc	Notes
Pedersen and Nielsen	[1]	1997	65	82		65	82		
Salzberg	[2]	1997	74	68		74	68		
Zien *et al.*	[3]	2000	76	78		76	78		
Zeng *et al.*	[4]	2002	76	94	85	76	94	85	
Pertea and Salzberg	[5]	2002			84			84	
Sayes *et al.*	[6]	2007	80	81		80	81		
Tikole	[7]	2008	83	73	74	83	73	74	
iTIS-PseTNC	[8]	2014						78	N1
TITER	[9]	2017				81	90	85	N2
DeepGSR	[10]	2018			94			94	
Goel *et al*.	[11]	2020	77	98	97	77	98	97	N3

Table 1: Performance comparison between different genomic TIS location prediction tools. Se denotes sensitivity, Sp specificity and Acc accuracy.

Notes

N1: tested in [10] on genomic data

N2: tested in [10] on genomic data

N3: Reported results from Table 2 in [11]

References

[1] A. G. Pedersen and H. Nielsen, "Neural network prediction of translation initiation sites in eukaryotes: perspectives for EST and genome analysis.," *Proceedings. Int. Conf. Intell. Syst. Mol. Biol.*, vol. 5, pp. 226–233, 1997.

[2] S. L. Salzberg, "A method for identifying splice sites and translational start sites in eukaryotic mRNA.," *Comput. Appl. Biosci.*, vol. 13, no. 4, pp. 365–376, Aug. 1997, doi: 10.1093/bioinformatics/13.4.365.

[3] A. Zien, G. Rätsch, S. Mika, B. Schölkopf, T. Lengauer, and K. R. Müller, "Engineering support vector machine kernels that recognize translation initiation sites.," *Bioinformatics*, vol. 16, no. 9, pp. 799–807, Sep. 2000, doi: 10.1093/bioinformatics/16.9.799.

[4] F. Zeng, R. H. C. Yap, and L. Wong, "Using feature generation and feature selection for accurate prediction of translation initiation sites.," *Genome Inform.*, vol. 13, pp. 192–200, 2002.

[5] M. Pertea and S. L. Salzberg, "A Method to Improve the Performance of Translation Start Site Detection and Its Application for Gene Finding," in *Algorithms in Bioinformatics*, 2002, pp. 210–219.

[6] Y. Saeys, T. Abeel, S. Degroeve, and Y. Van de Peer, "Translation initiation site prediction on a genomic scale: Beauty in simplicity," *Bioinformatics*, vol. 23, no. 13, pp. i418–i423, Jul. 2007, doi: 10.1093/bioinformatics/btm177.

[7] S. Tikole and R. Sankararamakrishnan, "Prediction of translation initiation sites in human mRNA sequences with AUG start codon in weak Kozak context: A neural network approach," *Biochem. Biophys. Res. Commun.*, vol. 369, no. 4, pp. 1166–1168, 2008, doi: https://doi.org/10.1016/j.bbrc.2008.03.008.

[8] W. Chen, P.-M. Feng, E.-Z. Deng, H. Lin, and K.-C. Chou, "iTIS-PseTNC: A sequence-based predictor for identifying translation initiation site in human genes using pseudo trinucleotide composition," *Anal. Biochem.*, vol. 462, pp. 76–83, 2014, doi: https://doi.org/10.1016/j.ab.2014.06.022.

[9] S. Zhang, H. Hu, T. Jiang, L. Zhang, and J. Zeng, "TITER: predicting translation initiation sites by deep learning.," *Bioinformatics*, vol. 33, no. 14, pp. i234–i242, Jul. 2017, doi: 10.1093/bioinformatics/btx247.

[10] M. Kalkatawi, A. Magana-Mora, B. Jankovic, and V. B. Bajic, "DeepGSR: an optimized deep-learning structure for the recognition of genomic signals and regions," *Bioinformatics*, vol. 35, no. 7, pp. 1125–1132, Apr. 2019, doi: 10.1093/bioinformatics/bty752.

[11] N. Goel, S. Singh, and T. C. Aseri, "Global sequence features based translation initiation site prediction in human genomic sequences.," *Heliyon*, vol. 6, no. 9, p. e04825, Sep. 2020, doi: 10.1016/j.heliyon.2020.e04825.
