# Supplementary material for: From shallow to deep: some lessons learned from application of machine learning for recognition of functional genomic elements in human genome
Source: Hum Genomics. 2022 Feb 18;16:7. doi: 10.1186/s40246-022-00376-1 (PMC8855580; doi:10.1186/s40246-022-00376-1)
Supplement: Supplementary file 2 — Additional file 2. Supplementary material 2. Table 1: Performance comparison between splice site prediction tools. Se denotes sensitivity, Sp specificity and Acc accuracy. [file 40246_2022_376_MOESM2_ESM.pdf]

SUPPLEMENTARY MATERIAL 2

The table below lists the results of surveyed prediction tools for locations in genome corresponding to splice sites. Table gives reported, retested and adjusted performance metrics. Where tools are not named by their authors, the name of the first author is used. For each tool, the first row is the performance on acceptor site and the second on donor sites.

Tool	Reference	Year	Reported			Retest 1				Adjusted Values		
			Se	Sp	Acc	Se	Sp	Acc	Notes	Se	Sp	Acc
GeneSplicer	[1]	2001				69	97	83	N1	69	97	83
						60	99	79		60	99	79
SplicePredictor	[2]	2004				84	92	88	N2	84	92	88
						79	97	88		79	97	88
Zhang	[3]	2010				90	90		N3	90	90	
						93	93			93	93	
Bari	[4]	2012	75	85	93	79	93	87	N4	77	89	89
			88	97	95	89	97	95		89	97	95
Goel	[5]	2015	94	94						94	94	
			91	94						91	94	
Wen	[6]	2017			93							93
					92							92
DeepSS	[7]	2018						95	N5			95
								95				95
SpliceRover	[8]	2018	91	97	96	91	97	95	N6	91	97	95
			90	97	95	90	96	96		90	96	96
Splice2Deep	[9]	2020	98	95	97					98	95	97
			99	96	97					99	96	97

Table 1: Performance comparison between splice site prediction tools. Se denotes sensitivity, Sp specificity and Acc accuracy.

Notes

N1: Tested in [9]

N2: Tested in [9] on current version of the prediction tool

N3: Averaged reported values for different model configurations

N4: Reported and calculated by the authors; retested in [8]

N5: Tested in [9]

N6: Retested in [9]

References

[1] M. Pertea, X. Lin, and S. L. Salzberg, "GeneSplicer: a new computational method for splice site prediction," *Nucleic Acids Res.*, vol. 29, no. 5, pp. 1185–1190, Mar. 2001.

[2] V. Brendel, L. Xing, and W. Zhu, "Gene structure prediction from consensus spliced alignment of multiple ESTs matching the same genomic locus.," *Bioinformatics*, vol. 20, no. 7, pp. 1157–1169, May 2004.

[3] Q. Zhang, Q. Peng, Q. Zhang, Y. Yan, K. Li, and J. Li, "Splice sites prediction of Human genome using length-variable Markov model and feature selection," *Expert Syst. Appl.*, vol. 37, no. 4, pp. 2771–2782, 2010.

[4] B.-S. Bari, Golam, Reaz, Rokeya, Jeong, "Effective DNA encoding for splice site prediction using SVM," *MATCH Commun. Math. Comput. Chem*, vol. 4, no. 4, pp. 1–125, 2012.

[5] N. Goel, S. Singh, and T. C. Aseri, "An Improved Method for Splice Site Prediction in DNA Sequences Using Support Vector Machines," *Procedia Comput. Sci.*, vol. 57, pp. 358–367, 2015.

[6] W. Li, J. Li, L. Huo, W. Li, and X. Du, "Prediction of Splice Site Using Support Vector Machine with Feature Selection," in *Proceedings of the International Conference on Bioinformatics and Computational Intelligence*, 2017, pp. 1–5.

[7] X. Du, Y. Yao, Y. Diao, H. Zhu, Y. Zhang, and S. Li, "DeepSS: Exploring Splice Site Motif Through Convolutional Neural Network Directly From DNA Sequence," *IEEE Access*, vol. 6, pp. 32958–32978, 2018.

[8] J. Zuallaert, F. Godin, M. Kim, A. Soete, Y. Saeys, and W. De Neve, "SpliceRover: interpretable convolutional neural networks for improved splice site prediction," *Bioinformatics*, vol. 34, no. 24, pp. 4180–4188, Dec. 2018.

[9] S. Albaradei *et al.*, "Splice2Deep: An ensemble of deep convolutional neural networks for improved splice site prediction in genomic DNA," *Gene X*, vol. 5, p. 100035, 2020.
