# Supplementary material for: From shallow to deep: some lessons learned from application of machine learning for recognition of functional genomic elements in human genome
Source: Hum Genomics. 2022 Feb 18;16:7. doi: 10.1186/s40246-022-00376-1 (PMC8855580; doi:10.1186/s40246-022-00376-1)
Supplement: Supplementary file 3 — Additional file 3. Supplementary material 3. Table 1: Performance comparison between different poly(A) tail prediction tools. Se denotes sensitivity, Sp specificity and Acc accuracy. [file 40246_2022_376_MOESM3_ESM.pdf]

SUPPLEMENTARY MATERIAL 3

The table below lists the surveyed Poly(A) tail location prediction tools and details reported and reassessed performances. Certain tools are not named by their authors in which case we used first author's name.

Tool	Reference	Year	Reported			Retest 1				Retest 2				Adjusted Values		
			Se	Sp	Acc	Se	Sp	Acc	Notes	Se	Sp	Acc	Notes	Se	Sp	Acc
Polyadq	[1]	1999				28	84	56	n1	63	88	74	n2	46	86	65
PolyA Signal Miner	[2]	2003	56-89	68-93										72	80	
ERPIN	[3]	2003	56	69-85		66	88	75	n3					66	88	75
PolyA_SVM	[4]	2006	37-71	75-97		58	64	61	n4	87	24	56	n5	56	78	68
PolyFd/PolyFud	[5]	2009	58-83	73-86	73-85	72	80	78	n6					72	80	78
Polyapred	[6]	2009	57	76-96										57	86	
Polyar	[7]	2010	24-95	15-66		57	50	53	n7	71	45	58	n8	57	50	53
Chang *et al.*	[8]	2011	56			56	90	75	n9					56	90	75
DPS-ANN	[9]	2012	81	84	82			74	n10							78
HMM-SVM	[10]	2013	84	87	85			75	n11	76	87	81	n12	80	87	81
DSET	[11]	2015	86	86	86				n13					86	86	86
Omni_PolyA	[12]	2018			88			76	n14			76	n15			80
DeepGSR	[13]	2019			84											84
DeeReCT-PolyA	[14]	2019			90			77	n16							84

Table 1: Performance comparison between different poly(A) tail prediction tools. Se denotes sensitivity, Sp specificity and Acc accuracy.

Notes

n1: Retest 1 in [9] using DPS dataset

n2: Retest 2 in [5]

n3: Retest 1 in [5]

n4: Retest 1 in [9]

n5: Retest 2 in [11]

n6: Retest 1 is calculated from data supplied in [5]

n7: Retest 1 in [9]

n8: Retest 2 in [11]

n9: Retest 1 recalculated from data supplied in [8]

n10: Retest 1 in [12]

n11: Retest 1 in [12]; midpoint of tests on two datasets ([DPS and GENCODE)

n12: Retest 2 in [11]

n13: Reported for DSET, combination of HMM_SVM, RF, PCA-SVM Oligo-string kernels

n14: Retest 1 on GENCODE dataset

n15: Retest 2 [14] DeeReCT-PolyA

n16: Reported on DPS data, retest 1 on GENCODE

References

[1] J. E. Tabaska and M. Q. Zhang, “Detection of polyadenylation signals in human DNA sequences.,” *Gene*, vol. 231, no. 1–2, pp. 77–86, Apr. 1999, doi: 10.1016/s0378-1119(99)00104-3.

[2] H. Liu, H. Han, J. Li, and L. Wong, “An in-silico method for prediction of polyadenylation signals in human sequences.,” *Genome Inform.*, vol. 14, pp. 84–93, 2003.

[3] M. Legendre and D. Gautheret, “Sequence determinants in human polyadenylation site selection.,” *BMC Genomics*, vol. 4, no. 1, p. 7, Feb. 2003, doi: 10.1186/1471-2164-4-7.

[4] Y. Cheng, R. M. Miura, and B. Tian, “Prediction of mRNA polyadenylation sites by support vector machine,” *Bioinformatics*, vol. 22, no. 19, pp. 2320–2325, Oct. 2006, doi: 10.1093/bioinformatics/btl394.

[5] M. Kamasawa and J.-I. Horiuchi, “Prediction of non-canonical polyadenylation signals in human genomic sequences based on a novel algorithm using a fuzzy membership function.,” *J. Biosci. Bioeng.*, vol. 107, no. 5, pp. 569–578, May 2009, doi: 10.1016/j.jbiosc.2009.01.001.

[6] F. Ahmed, M. Kumar, and G. P. S. Raghava, “Prediction of polyadenylation signals in human DNA sequences using nucleotide frequencies.,” *In Silico Biol.*, vol. 9, no. 3, pp. 135–148, 2009.

[7] M. N. Akhtar, S. A. Bukhari, Z. Fazal, R. Qamar, and I. A. Shahmuradov, “POLYAR, a new computer program for prediction of poly(A) sites in human sequences.,” *BMC Genomics*, vol. 11, p. 646, Nov. 2010, doi: 10.1186/1471-2164-11-646.

[8] T.-H. Chang *et al.*, “Characterization and prediction of mRNA polyadenylation sites in human genes,” *Med. Biol. Eng. Comput.*, vol. 49, no. 4, pp. 463–472, 2011, doi: 10.1007/s11517-011-0732-4.

[9] M. Kalkatawi *et al.*, “Dragon PolyA Spotter: predictor of poly(A) motifs within human genomic DNA sequences.,” *Bioinformatics*, vol. 28, no. 1, pp. 127–129, Jan. 2012, doi: 10.1093/bioinformatics/btr602.

[10] B. Xie, B. R. Jankovic, V. B. Bajic, L. Song, and X. Gao, “Poly(A) motif prediction using spectral latent features from human DNA sequences,” *Bioinformatics*, vol. 29, no. 13, pp. i316–i325, Jul. 2013, doi: 10.1093/bioinformatics/btt218.

[11] S. Zhang, J. Han, J. Liu, J. Zheng, and R. Liu, “An improved poly(A) motifs recognition method based on decision level fusion.,” *Comput. Biol. Chem.*, vol. 54, pp. 49–56, Feb. 2015, doi: 10.1016/j.compbiolchem.2014.12.001.

[12] A. Magana-Mora, M. Kalkatawi, and V. B. Bajic, “Omni-PolyA: a method and tool for accurate recognition of Poly(A) signals in human genomic DNA,” *BMC Genomics*, vol. 18, no. 1, p. 620, 2017, doi: 10.1186/s12864-017-4033-7.

[13] M. Kalkatawi, A. Magana-Mora, B. Jankovic, and V. B. Bajic, “DeepGSR: an optimized deep-learning structure for the recognition of genomic signals and regions,” *Bioinformatics*, vol. 35, no. 7, pp. 1125–1132, Apr. 2019, doi: 10.1093/bioinformatics/bty752.

[14] Z. Xia *et al.*, “DeeReCT-PolyA: a robust and generic deep learning method for PAS identification,” *Bioinformatics*, vol. 35, no. 14, pp. 2371–2379, Jul. 2019, doi: 10.1093/bioinformatics/bty991.
